# Mechanosensing in Dendritic Cells

**DOI:** 10.1111/imr.70086

**Published:** 2025-12-14

**Authors:** Vincent Calmettes, Melissa A. Quintanilla, Livia Lacerda Mariano, Matthieu Piel, Hélène D. Moreau, Ana‐Maria Lennon‐Duménil

**Affiliations:** ^1^ Institut Curie PSL Research University, INSERM U932 Paris France; ^2^ Institut Curie and Institut Pierre Gilles de Gennes PSL Research University, CNRS UMR 144 Paris France

**Keywords:** cell confinement, cell migration, cytoskeleton, dendritic cells, extracellular matrix, mechanosensing, nucleus, rigidity, tissue physical properties

## Abstract

Since their discovery, dendritic cells have been recognized for their unusual capacity to sense and respond to physical stimuli within their environment. However, it took nearly two decades—and the advent of mechanobiology—to elucidate the underlying mechanisms and functional implications of this mechanical hypersensitivity. In this review, we first outline the fundamental principles by which cells interact with their physical surroundings and transduce mechanical cues into biological responses. We then examine these concepts in the context of dendritic cell biology, highlighting how mechanosensing shapes their immune phenotype and governs their migratory behavior across tissues. Emerging evidence reveals that dendritic cells possess remarkable adaptability to mechanical constraints, a property that critically defines their role in immune surveillance. These insights underscore the need to consider mechanical cues as key regulators of dendritic cell function, particularly in pathological settings where tissue mechanics are altered, such as cancer and fibrosis‐associated autoimmune diseases.

## 1 Introduction

The emerging field of mechanobiology has led to growing interest in how cells respond to physical cues—specifically, how mechanical forces and the physical properties of the cell and tissue microenvironment influence biological systems. This includes how cells detect mechanical stimuli (mechanosensing), convert them into biochemical signals (mechanotransduction), and adapt their behavior and function accordingly. Seminal studies showed that stem cells cultured in the presence of the same growth factors undergo drastically distinct differentiation programs when exposed to environments of different rigidities [1]. During embryogenesis, mechanical forces guide key developmental processes, including germ layer specification, axis formation, and coordinated tissue folding [2]. For example, actomyosin contractility and supracellular pulling forces integrate morphogen gradients and orchestrate cell rearrangements and fate decisions, ensuring robust and reproducible patterning of the embryo. The relevance of mechanobiology extends to pathology, particularly in cancer, where remodeling of the extracellular matrix (ECM) and increased tissue stiffness can induce epithelial‐to‐mesenchymal transition (EMT), impair immune response and promote metastasis [3]. These observations highlight the universal importance of mechanical cues across diverse biological contexts.

A great amount of work in immunology focused on how biochemical factors, such as cytokines and chemokines, regulate the steps involved in the initiation and resolution of inflammation. Increasing evidence, nonetheless, suggests that the way immune cells sense and respond to environmental mechanical properties is also crucial to mount an effective immune response. Neutrophils, for example, activate Piezo1, a mechanosensitive calcium channel, while deforming as they transmigrate between the endothelial cells from the blood to the tissue. The activation of Piezo1 upregulates the expression of nicotinamide adenine dinucleotide phosphate (NADPH) oxidase 4 (Nox4), increasing their capacity to kill bacteria in tissues [4]. Similarly, macrophages, which are tissue resident cells, can sense tissue stiffness. When contacting a soft substrate, they polarize into a pro‐inflammatory phenotype, whereas on stiffer substrates they acquire an elongated morphology and polarize into an anti‐inflammatory phenotype prone to tissue regeneration [5]. Interestingly, stiff tumors benefit from this stiffness‐sensing capacity of macrophages to evade immune killing. Indeed, in stiff fibrotic tumors, macrophages synthesize collagen proteins, which requires the consumption of environmental amino acids that are then no longer available for cytotoxic CD8^+^ T cells [6]. In late‐stage tumors, the depletion of macrophages is also sufficient to activate a fibrotic response in tumor cells, increasing collagen 3 deposition and immune cell infiltration [7]. Mechanosensing can further impact the ability of macrophages to selectively phagocyte targets such as microbes or cell‐derived vesicles that display distinct physical characteristics [8, 9]. Finally, substrate rigidity enhances activation and cytokine production in T cells [10, 11]. This might constitute a checkpoint for the onset of adaptive immune responses as the antigen presenting cells T cells interact with (macrophages and dendritic cells (DCs)) display a wide range of rigidities depending on the environmental inflammatory conditions [12]. These findings illustrate the variety of immune processes that are impacted by the physical properties of cells and tissues, modifying the outcome of immune responses against infection and cancer.

This might particularly apply to DCs given their ability to migrate across tissues and organs exhibiting variable mechanical properties [13, 14]. At steady state, DCs explore the interstitial space of tissues by combining random movements with the uptake of extracellular material [15, 16]. Upon encountering a danger signal, they enter a “maturation program,” which downregulates internalization and stimulates their fast migration to lymph nodes for presentation of the collected antigens to T lymphocytes [17]. Importantly, all the processes needed for DCs to achieve their immune‐surveillance function—cell migration, antigen uptake, cell maturation, and antigen presentation—depend on the inflammatory state and physical properties of tissues, in addition to the nature of antigens themselves, and will determine the type of T‐cell response ultimately developed. While most of the published work over decades has focused on chemical signals (damage‐ and pathogen‐associated molecular patterns, chemokines) that DCs encounter in tissues, the role of physical cues has only recently begun to be appreciated.

In this review, we discuss our current understanding of mechano‐regulation in DCs: how environmental physical properties shape their capacity to patrol the environment and mature, and what the consequences of these regulatory processes are on adaptive immune responses. We further discuss recently uncovered mechanisms used by DCs to sense and transduce mechanical signals, which involve not only their cytoskeleton but also their nucleus, which has emerged as a major player in the mechanobiology field.

## 2 The Cytoskeleton Enables Dendritic Cells to Interact and Sense Their Mechanical Environment

### 2.1 Extracellular Matrix Organization and Tissue Physical Constraints in Health and Disease

**FIGURE 1:**
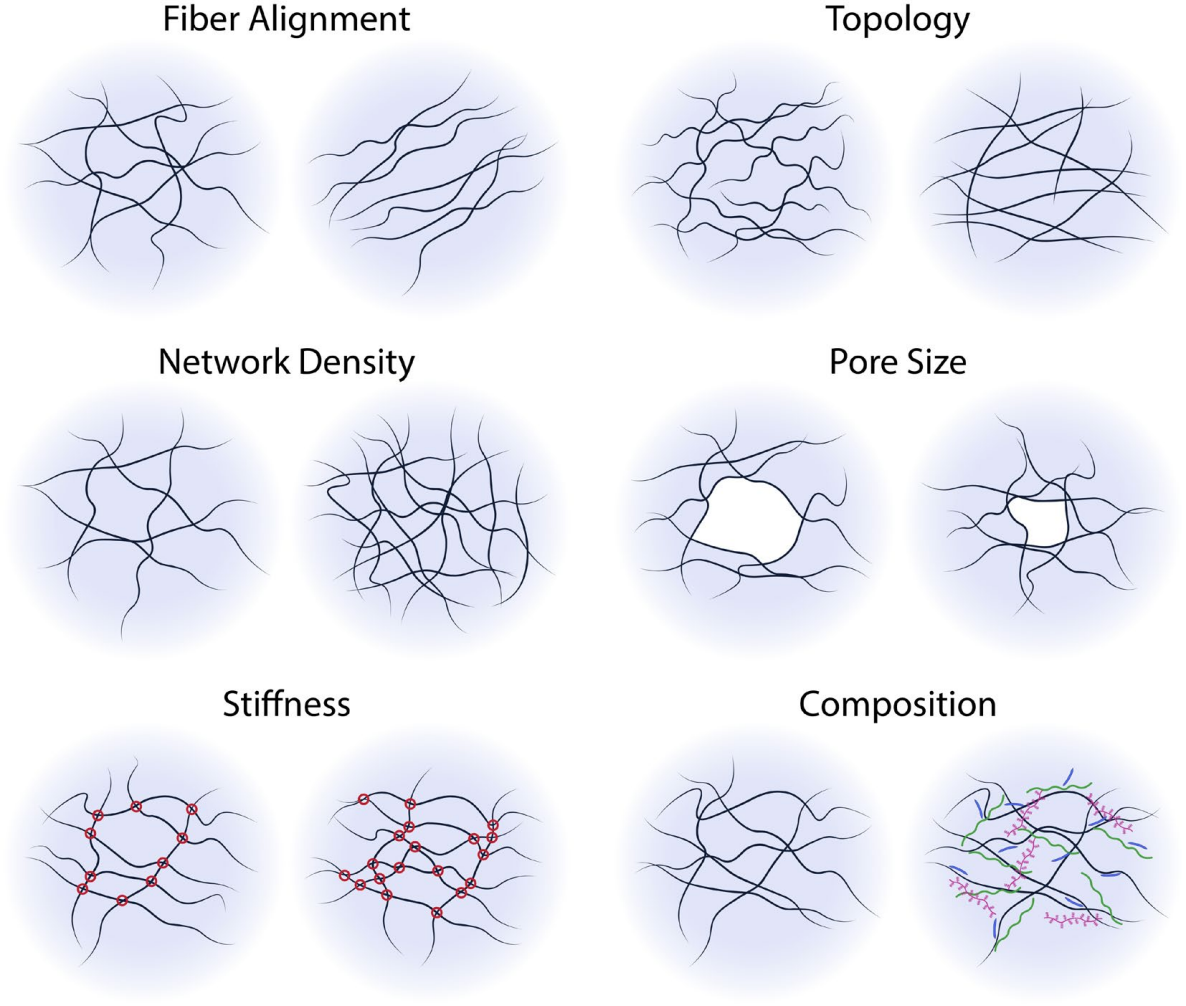
Organization and properties of the extracellular matrix. Illustration of the structural organization and biophysical properties of the extracellular matrix that influence dendritic cell (DC) motility and behavior within tissues. The ECM is composed of a complex network of fibers and macromolecules whose arrangement varies across different tissue types. Fiber alignment, topology, and network density collectively determine the pore size that migrating DCs encounter, thereby shaping the paths available for their movement. Increased crosslinking of collagen fibers enhances tissue stiffness, which restricts the ability of cells to deform the ECM. Beyond its mechanical characteristics, the ECM is also defined by its molecular composition, including collagens, elastin, proteoglycans, and glycoproteins. These components not only confer distinct physical properties to the tissue but also regulate the local biochemical environment by modulating the availability, distribution, and solubility of signaling molecules. Together, these structural and compositional features of the ECM create a dynamic landscape that governs how DCs navigate, adapt, and perform their immune functions within diverse tissue contexts.

Pre‐DCs exit the bloodstream and enter non‐lymphoid tissues where they become immature DCs (iDCs) in charge of environment patrolling. Once mature (mDCs), they migrate to lymph nodes where they activate T cells and initiate the adaptive immune response. DCs are therefore strategically distributed throughout most organs and tissues; hence, they are exposed to a variety of physical constraints imposed by the composition and organization of the tissue extracellular matrix (ECM). The ECM is a dynamic network that constitutes the skeleton of tissues. ECM remodeling is a highly dynamic and continuous process essential in regulating immune responses [18] and maintaining tissue homeostasis [19]. It involves not only the degradation of matrix components by enzymes such as matrix metalloproteinases, cathepsins, and hyaluronidases, but also the synthesis of new ECM elements by fibroblasts, chondrocytes and osteoblasts, and many other cell types, including macrophages [20]. This remodeling is essential for normal tissue repair, morphogenesis, and adaptation to physiological and pathological conditions, but its dysregulation underlies the pathogenesis of many diseases, including cancer and fibrosis [21]. The occurrence of such pathologies also modifies the ECM [3]. In cancer, aberrant ECM remodeling leads to increased matrix stiffness, altered architecture, and changes in the abundance of bioactive molecules, which collectively promote tumor cell invasion, immune evasion, and resistance to therapy [22]. In fibrosis, excessive ECM deposition and reduced degradation result in tissue stiffening and impaired organ function, with fibroblast‐immune cell interactions driving pathological ECM accumulation. The physical properties of the tissues therefore evolve, and impose different constraints on DCs in health and disease, as described below (Figure 1).

– *The geometry of ECM*—including fiber alignment, pore size, and network organization—dictates the degree of cell confinement and the available migratory paths. ECM geometries range from highly organized fibrillar networks (e.g., collagen fibers in tendon) to more amorphous gel‐like structures (e.g., basement membrane in epithelia).
– *The chemical composition* of the ECM is tissue‐specific, comprising around 300 proteins, including laminins, collagens (providing tensile strength), elastin (providing elasticity), proteoglycans, glycosaminoglycans (GAGs), and glycoproteins (providing resistance to compression). Notably, chemokines, such as CCL21 [23, 24], can bind to GAGs. This composition changes under inflammatory conditions, which in turn regulates the way cells interact with the ECM.
– *Mechanical properties*—notably stiffness and elasticity—vary widely between tissues. Organ stiffness varies from hundreds of Pascals (Pa) in brain/bone marrow to 10 kPa in muscle, and more than 100 kPa in bone.
– *Hydrostatic pressure* is shaped by vascular permeability, interstitial fluid production, and lymphatic drainage. Interstitial pressure increases in inflamed or highly vascularized tissues, as well as in tumors.

The strategies employed by DCs to patrol their environment will in part be determined by these tissue physical properties, which are tightly regulated in a dynamic and tissue‐specific manner upon organ infection or injury (sterile inflammation, cancer). Investigating the mechanisms by which DCs sense these properties and the consequences of such sensing on their ability to dialog with T cells is thus essential to understand how tissue‐specific physical cues define the outcome of adaptive immune responses.

### 2.2 The Dendritic Cell Cytoskeleton: a Mechanosensing Machinery

**FIGURE 2:**
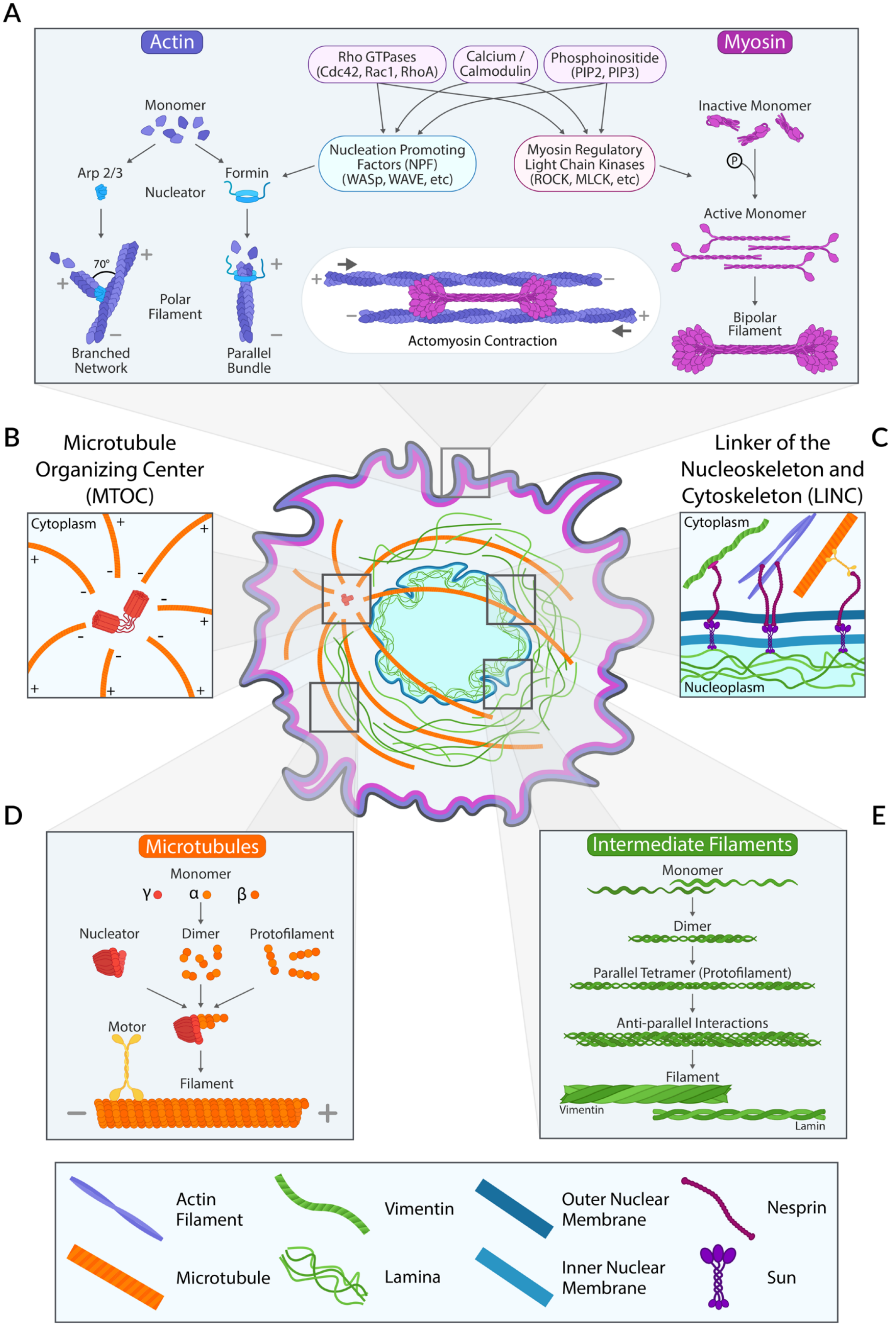
Cytoskeletal machinery regulating dendritic cell responses to ECM physical properties. The actomyosin cytoskeleton (A) is a central driver of dendritic cell migration. Spatial regulation of actin polymerization and acto‐myosin contractility is mediated by Rho GTPases, calcium influx, and phosphoinositide signaling. These cues activate nucleation‐promoting factors (NPFs), such as WASp and WAVE, which stimulate actin nucleators to form branched (via Arp2/3 complex) or linear (via formins) filament networks. Myosin monomers polymerize into bipolar filaments that contract preassembled actin structures, enabling force generation. Microtubules are polarized filaments nucleated from the microtubule‐organizing center (MTOC), whose position adapts to spatial constraints (B). They serve as tracks for motor proteins (kinesin and dynein) that transport organelles and vesicles, contributing to intracellular organization and cell polarity (D). Intermediate filaments, including lamin and vimentin, provide structural support to the nucleus and maintain nuclear envelope integrity under mechanical stress (E). All three cytoskeletal systems interface with the nuclear envelope via the LINC (Linker of Nucleoskeleton and Cytoskeleton) complex, which connects SUN domain proteins on the inner nuclear membrane to nesprins on the outer membrane, facilitating mechanotransduction between the cytoskeleton and nucleus (C).

The cytoskeleton enables DCs to sense and respond to their physical environment by mechanically and biochemically coupling the cell to its surroundings, organizing internal components, determining shape, and facilitating motility [25]. As shown in Figure 2, it comprises actin filaments, microtubules, and intermediate filaments—protein polymers with distinct dynamics, polarity, stiffness, and motor protein interactions [26]. Motor proteins—actin‐binding myosins and microtubule‐binding kinesins and dyneins—organize filament networks and transport intracellular cargos. Post‐translational modifications of the cytoskeleton can also alter the function and physical properties of these filaments, a regulation mode particularly well‐defined for microtubules [27]. Cytoskeletal dynamics depend on numerous regulatory proteins that control filament polymerization, growth, stabilization, capping, and depolymerization. Spatiotemporal control of cytoskeletal organization and motor activity is governed by signaling pathways involving Rho GTPases (RhoA, Rac2, Cdc42), phosphoinositide, and calcium signaling [28]. Below, we provide a brief overview of the organization and dynamics of the distinct cytoskeletal networks, which collectively enable DCs to flexibly remodel their cytoskeletal architecture in response to environmental cues and functional demands.

#### 2.2.1 The Actomyosin Cytoskeleton

The actomyosin cytoskeleton (Figure 2A), composed of actin filaments and myosin motor proteins, plays a central role in DC biology by regulating dynamic network assembly and cellular mechanics [29], reviewed in detail by Rottner et al. [30]. Actin polymerization begins when monomeric G‐actin binds in a polar fashion, a process that can occur spontaneously but is primarily driven by nucleation proteins. Two key nucleators are the Arp2/3 complex, which generates branched actin networks, and Formins, which produce linear filaments. These nucleators are activated by nucleation‐promoting factors (NPFs) such as Wasp, Wave, and Wash, themselves regulated by Rho‐GTPases, which localize activity to specific cellular regions, leading to the formation of specific structures. Actin dynamics are further modulated by actin‐binding proteins (ABPs) like profilin, cofilin, Ena/VASP, and capping proteins. Notably, mutations in the *Wasp* gene cause Wiskott‐Aldrich Syndrome, a severe immunodeficiency, while mutations in *Arpc1b*, a subunit of Arp2/3, lead to similar pathologies, underscoring the importance of actin regulation in immune function.

**FIGURE 3:**
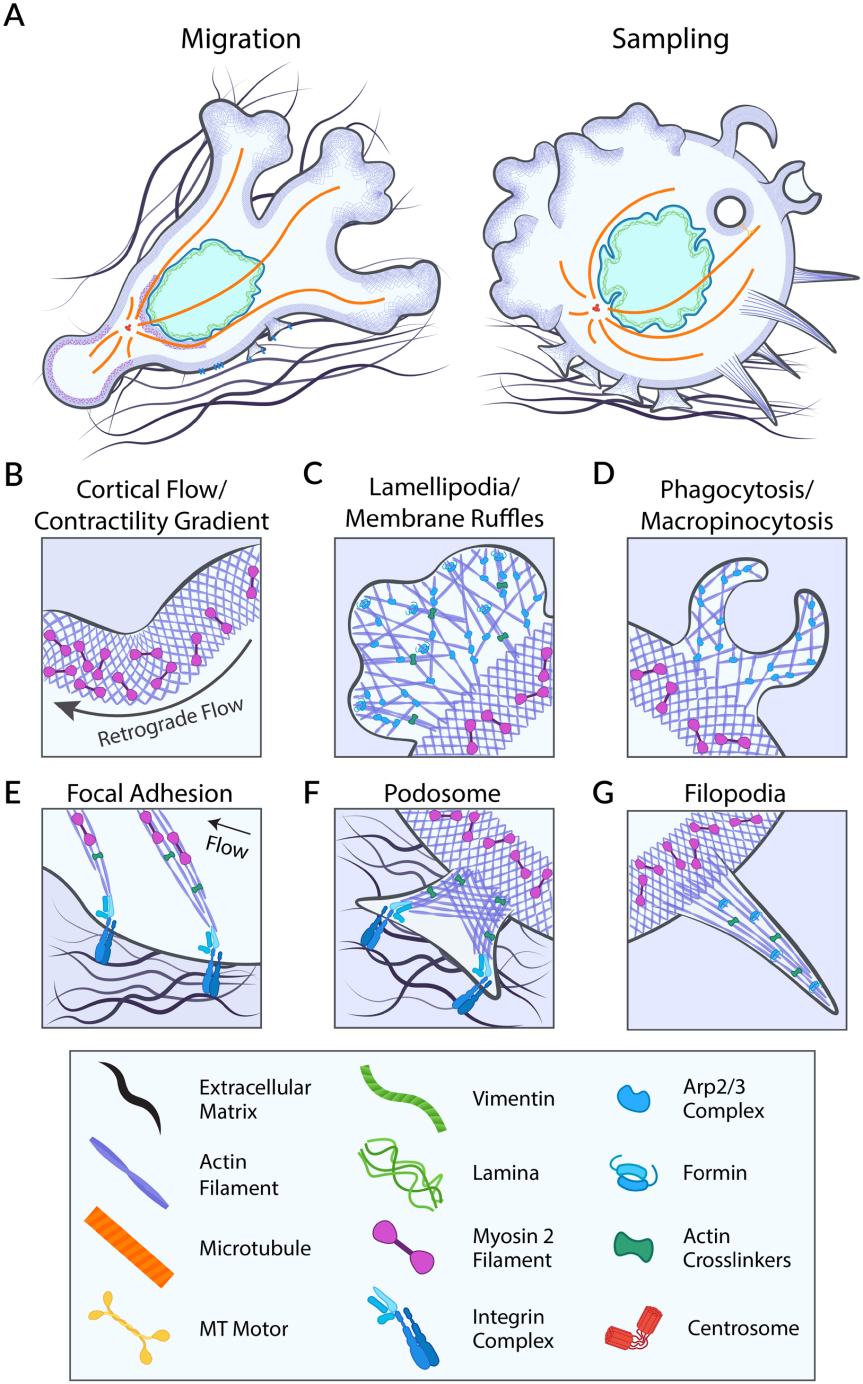
Cytoskeletal dynamics orchestrate dendritic cell function. iDCs alternate between phases of rapid migration and periods of slow migration coupled with macropinocytosis, while mDCs adopt a fast and persistent migration mode (A). In confined environments, DCs rely on retrograde cortical actomyosin flow to generate integrin‐independent friction against the extracellular matrix (ECM), enabling rapid amoeboid migration (B). At the leading edge, Arp2/3‐driven branched actin networks promote lamellipodia formation, which probes ECM architecture, expands spatial exploration and enhances migratory capacity (C). These networks also give rise to membrane ruffles that support macropinocytosis (D). DCs can also engage the ECM through integrin‐dependent adhesion complexes such as focal adhesions and podosomes, resulting in slower migration and localized ECM remodeling via secretion of metalloproteases (E and F). Filopodia—stabilized by Formin‐mediated parallel actin bundles—serve as sensory structures that probe ECM organization and support directional migration (G). Together, these cytoskeletal structures and adhesion mechanisms form a highly dynamic and interconnected system that allows DCs to integrate mechanical and biochemical cues from the ECM, and coordinate migration with immune surveillance functions.

Actin can assemble into diverse cellular structures (Figure 3), including protrusions and cortical networks, which enable cells to sense and adapt to extracellular constraints. Actin‐based protrusions include blebs, lamellipodia, and filopodia. Blebs form when Myosin II‐driven contraction detaches the membrane from the cortex [31], often in response to cell confinement [32]. Lamellipodia, composed of branched and bundled actin downstream of Rac1 and Cdc42, form at adhesive interfaces or as dorsal ruffles. These Rho‐GTPases enable phagocytosis and macropinocytosis, respectively. Filopodia, built from bundled actin filaments, extend from the cortex, probe the ECM and sense chemokines guiding DC migration.

Cortical actin, located beneath the plasma membrane, is anchored via membrane‐to‐cortex attachment proteins from the ERM family (Ezrin, Radixin, Moesin) [33]. The actin cortex plays a central role in defining cell shape and surface tension [34]. This dense meshwork, nucleated by Arp2/3 and Formins, incorporates actin crosslinkers and Myosin II motors, whose penetration influences cortical mechanics [35]. Asymmetric cortical tension and rear contraction generate retrograde actin flow, particularly within protrusive structures [32, 36, 37, 38]. This cortical layer is essential for maintaining cell shape and mediating adhesion to the ECM through integrins. Integrin‐actin coupling forms specialized adhesion structures, including focal adhesions and podosomes, the latter being particularly prominent in DCs cultured on 2D surfaces. Additionally, cortical actin connects to the nucleus via the LINC complex (Figure 2C), which links the cytoskeleton to the nuclear lamina [39]. Other ABPs, such as Fascin and Filamin, crosslink filaments and form structures like filopodia—thin protrusions that guide cell migration through the extracellular matrix (ECM). ABPs also mediate interactions between actin and other cytoskeletal elements, such as microtubules and intermediate filaments.

Actin networks play a direct role as mechanosensors, as different physical constraints impact their organization and dynamics. For example, branched actin networks respond to increased mechanical load by enhancing actin polymerization and crosslinking, which results in network densification and stiffening [40, 41, 42]. However, if mechanical load keeps increasing, branched actin networks start softening from filament buckling or crosslinker rearrangement [43]. This mechanical adaptation affords mechanical resilience to cells, a role that also involves intermediate filament networks [44, 45]. Myosin II, a non‐muscle contractile motor protein, is a key regulator of actin network tension and organization. It binds to actin filaments and uses ATPase‐driven power strokes to move toward filament plus ends. When assembled into bipolar filaments, Myosin II induces contraction by sliding anti‐parallel actin filaments together. This contractility not only stiffens the actin network but also influences filament turnover, flow, and force generation, as reviewed by others [46, 47] Myosin II activity is regulated by the kinases MLCK (calcium‐activated myosin light chain kinase) and ROCK (Rho‐associated Coiled‐coil Kinase) [48, 49, 50], which are activated downstream of the small GTPases Cdc42 and RhoA, whose signaling pathways can function antagonistically to fine‐tune cytoskeletal dynamics.

#### 2.2.2 Microtubules

Microtubules (Figure 2D) are helical polymers of α/β‐tubulin heterodimers forming a polarized lattice, initiated by nucleators like the γ‐tubulin ring complex (γ‐TURC), which templates 13 protofilaments. This creates a dynamic plus end and a more stable minus end. Polarity enables intracellular cargo transport via motor proteins: kinesins move toward plus ends, dyneins toward minus ends. Microtubules exhibit dynamic instability, alternating between growth and depolymerization phases [51]. They are organized by the microtubule organizing center (MTOC, Figure 2B), or centrosome [52], whose position varies by cell type: in mesenchymal cells (e.g., macrophages), it lies anterior to the nucleus, while in amoeboid cells (e.g., lymphocytes, dendritic cells), it varies depending on the ECM structure [53, 54]. Microtubule dynamics influence cell migration and mechanosensing (see Part 4). Notably, increasing evidence highlights crosstalk between microtubules and the actin cytoskeleton, as reviewed by Pimm and Henty‐Ridilla [55]. This interplay underscores the cytoskeleton's role in spatial organization and cellular behavior.

#### 2.2.3 Intermediate Filaments

Intermediate filaments (Figure 2E) are stable, apolar polymers that play diverse roles in cellular architecture and function, largely through interactions with other intracellular proteins (reviewed by Coelho‐Rato et al. [56]). They share a conserved alpha‐helical domain that dimerizes to form tetrameric protofilaments, which assemble laterally into tubular networks of variable diameter and length. In DCs, the primary intermediate filaments are vimentin and nuclear Lamins. Vimentin filaments contribute to mechanical resistance and mechanosensation, particularly in response to extracellular stress, by interacting with other cytoskeletal components [57, 58, 59]. Nuclear Lamins form a network that maintains nuclear shape, mechanical integrity, and organization of nuclear contents [60]. Lamin A (and its splice variant Lamin C) is developmentally regulated and expressed in a tissue‐ and differentiation‐dependent manner, with higher levels in differentiated and mechanically stressed cells such as muscle, whereas Lamins B1 and B2 are ubiquitously expressed [61]. They also differ in their mechanical roles: type A Lamins enhance nuclear viscosity, while type B Lamins increase elasticity [61]. Their relative expression modulates nuclear mechanics, especially under compression [62]. Lamins also connect the nucleus to the extracellular matrix via the LINC complex, which links SUN proteins on the inner nuclear membrane to nesprins on the outer membrane [39, 63]. Nesprins bind actin and microtubules, forming a physical bridge between the nucleoskeleton and cytoskeleton. Mechanical cues—such as nuclear compression during migration or tension from ECM–LINC interactions—regulate nuclear envelope tension. This tension influences chromatin organization, transcription factor localization, and gene expression [64]. The specific impact of Lamins and nuclear envelope tension on DC responses to ECM geometry is discussed below (Part 2).

## 3 Tissue Environment and ECM: A Dual Role in DC Migration

In complex three‐dimensional environments, the events of cell confinement experienced by DCs reflect the structural heterogeneity of the ECM. Such DC deformation events exert dual effects on their migration. First, they facilitate amoeboid motility by enabling DCs to generate frictional forces against surrounding ECM fibers. Second, they impose physical constraints that limit migration, requiring DCs to dynamically remodel their cytoskeleton and reposition intracellular organelles to navigate through narrow pores and dense ECM networks. Thus, ECM complexity not only provides the mechanical scaffold that supports DC locomotion but also acts as a selective barrier, shaping the strategies by which DCs adapt their migratory behavior to surrounding physical obstacles.

### 3.1 The Tissue Environment Promotes Amoeboid Migration by Providing Friction

Cell locomotion exhibits remarkable adaptability, with cells being able to transition between different migration modes. Immune cells can adopt either the mesenchymal migration mode or the amoeboid one depending on the environmental cues they are exposed to. Mesenchymal migration is characterized by elongated cell morphology, strong integrin‐mediated adhesions, and proteolytic remodeling of the ECM. In this case, forward movement strongly relies on actin polymerization at the cell front. In contrast, amoeboid migration features rounded cell shapes, weak or absent adhesions, and rapid forward movement driven by actomyosin contractility at the cell rear. Amoeboid migration also occurs independently of ECM degradation. As previously described [32], these two migration modes are complementary, dynamic, and reversible, and are primarily governed by the degree of cell adhesion and the physical constraints of the cell microenvironment.

The classical view of cell migration in the early 2000s was based on integrin‐mediated adhesion (mesenchymal migration): F‐actin polymerization at the cell front induces protrusion leading to adhesion by integrins to the substrate and actin retrograde flow. Actomyosin contraction allows cells pulling on adhesions, enabling a new protrusion to be initiated. This view was challenged by Lämmerman et al. [36] in 2008, who revealed that leukocytes can migrate rapidly through 3D environments independently of cell adhesion. Specifically, this study showed that mature DCs (mDCs) genetically ablated for key integrin genes or for the integrin adaptor *Talin1* retain their full migratory capacity within 3D collagen gels, as well as in vivo. Their migration was nonetheless impaired in 2D substrates. They found that the migration of these non‐adhesive leukocytes was primarily by actin network expansion at the leading edge and the retrograde actin flow that depends on actomyosin contractility. Later on, Renkawitz et al. [65] showed that mDCs can switch between integrin‐dependent and integrin‐independent migration in response to the adhesive properties of their environment. When integrins are engaged, the actin cytoskeleton is coupled to the substrate and actin polymerization is efficiently converted into forward migration. In contrast, when integrins are not engaged, cells compensate to maintain their speed by increasing the rate of retrograde flow, inducing friction forces. This migration mode is not restricted to mDCs, as immature DCs (iDCs) can also adopt amoeboid migration in confined and a low adhesive environment [32, 66]. These findings collectively redefined our understanding of immune cell motility, highlighting the adaptability of DCs to diverse tissue environments. It was proposed that the ability of these cells to switch between adhesion‐dependent and adhesion‐independent migration modes ensures efficient navigation through complex interstitial spaces and optimizes their immune surveillance function.

Key regulators of this cell migratory plasticity are the Rho‐family GTPase Cdc42, Rac, and RhoA, which regulate actin nucleating by Arp2/3 and formins. Rac1 and Rac2 are needed for mDCs to migrate toward the draining lymph node in vivo, and subsequent antigen presentation to T cells [67], most likely for the generation of cell protrusions that guide mDCs along CCL21 gradients. Cdc42 activity was reported to be crucial for maintaining front‐rear polarity of mDCs in 3D environments ex vivo and in vivo [68, 69]. Accordingly, Cdc42 knockout mDCs present multiple competitive leading edges and can rip off within tissues. No such defect is observed in DCs migrating within 1D or 2D environments. The Cdc42 GEF (guanine nucleotide exchange factor), Dock8, is also essential for mDC migration [70]. The molecular mechanisms accounting for this phenotype might involve both Arp2/3 and formins as both actin nucleators can interact with Cdc42 at the front of migrating cells [71]. These studies very well illustrate how the geometry of the ECM can differentially impact the mechanisms used by DCs to navigate within tissues.

As described above, DC ameboid migration is non‐destructive and does not strictly require adhesion to the ECM. Remarkably, Reversat et al. [72] found that leukocytes are capable of sensing changes within the topography of their microenvironment. When confined, these cells respond to textured or topographically complex substrates that can help propel themselves in less adhesive environments. As in amoeboid migration, this movement is driven by the retrograde flow of the actin cytoskeleton, which exerts pushing forces against environmental geometries, generating sufficient friction for forward propulsion. This process occurs without any detectable transmembrane force transmission to the ECM by transmembrane proteins, indicating that leukocytes can use purely mechanical interactions with their environment to migrate. This suggests that environmental geometry can substitute for biochemical adhesion in enabling cell motility. On the same line, Gaertner et al. [73] identified WASp as playing a critical role in the formation of mechanosensitive actin patches that enable immune cells to push against physical barriers and migrate effectively through confined spaces. Using 3D‐agarose gels of varying stiffness, they observed that increasing mechanical load reduced cell migration speed and increased arrest periods. Under intermediate stiffness, mDCs formed distinct actin‐rich patches scattered across the cell cortex when confined. These patches formed independently of Myosin II activity and cell adhesion, indicating a unique mechanism of force generation. They moved with the retrograde actin flow and occasionally evolved into knob‐like protrusions that extended vertically into the agarose gel, suggesting that they can exert pushing forces. Ultrastructural analysis confirmed that these knobs could indent the agarose gel and even deform the nuclear lamina, supporting their role in generating vertical forces. Importantly, nucleus‐free cytoplasts also formed actin patches and knobs under confinement, demonstrating that the nucleus is not required for this mechanosensitive response. The authors proposed that in contrast to WAVE, which generates horizontal protrusions, WASp helps DCs generate vertical protrusions. This study further identified Cip4, a curvature‐sensing F‐BAR domain protein, as a potential upstream regulator of WASp. Cip4 localized to ridges and preceded actin patch formation, suggesting that membrane curvature may signal for WASp activation.

Overall, these studies reveal that mDCs can use protrusions to respond to geometrical cues by transmitting forces to their environment and propelling themselves independently of cell adhesion.

### 3.2 The Tissue Environment Restricts Migration by Limiting DC Nucleus Passage

It is today clear that the intracellular organelle that predominantly limits cell deformation through narrow pores is the cell nucleus [74, 75]. The nucleus is the largest and the stiffest organelle in cells [62]. As described in Part 1, the relative abundance of Lamin A/C versus Lamin B modulates nuclear mechanical properties: cells with high Lamin A/C exhibit stiffer nuclei, whereas cells with low Lamin A/C have more deformable nuclei [76]. Decreased expression of Lamin A/C promotes cell migration as it allows cells to travel through physical obstacles upon nucleus deformation. However, this correlates with lower cell survival and increased DNA damage [75]. Unlike neutrophils, DCs express intermediate levels of Lamin A/C—higher than lymphocytes but lower than fibroblasts and metastatic cells—allowing them to balance survival with their migratory capacity.

Using microfabricated channels with defined constriction widths, Thiam et al. [77] observed that iDCs can pass through pores as narrow as 1.5 μm in diameter, but nuclear deformation becomes a limiting factor below 3 μm. Disruption of microtubules with Nocodazole enhances passage, while inhibition of Myosin II reduces cell speed but does not affect nuclear transit. In contrast, inhibition or depletion of Arp2/3 significantly impairs passage through narrow constrictions, without altering directionality or nuclear engagement. This indicates that Arp2/3 is specifically required for nuclear deformation, not general motility. Live imaging of LifeAct‐GFP DCs revealed a dense, transient accumulation of actin around the nucleus during constriction transit, termed the confinement‐induced actin network (CiAN). This perinuclear actin was spatially restricted to the constriction and temporally aligned with nuclear deformation. Its formation was independent of adhesion and did not require the LINC complex or even the nucleus itself, as enucleated cells still formed CiAN. Immunostaining confirmed that Arp3 co‐localized with perinuclear actin, while Myosin II‐GFP localized to the cell rear and was absent from the nuclear periphery. Blebbistatin treatment blocked Myosin accumulation at the rear but did not prevent CiAN formation, reinforcing the result showing that Arp2/3—but not Myosin II—is responsible for actin nucleation around the nucleus. Together, these findings define an Arp2/3‐dependent mechanism by which iDCs deform their nucleus to migrate through confined spaces, independent of adhesion and contractility, and crucially linked to perinuclear actin dynamics and transient Lamina rupture. Whether this actin structure observed around the nucleus when DCs are within confining microchannels is equivalent to the one observed in the perinuclear area of confined in 2D [78] which also relies on Arp2/3 activity, remains an open question.

Of note, a perinuclear actomyosin ring was also observed in mDCs migrating through 2 μm pores [79]. Phosphoproteomic analysis identified phosphorylation of cofilin‐1 at serine 41 as responsible for the formation of this ring. Cofilin binds to actin to promote filament severing and disassembly, which increases actin filament turnover. Phosphomimetic Colifin‐1 mutant DCs reproduce a similar pattern to mDCs, displaying an elongated nucleus and increased migration in 3D‐collagen gels. The integration of mechanical sensing, cytoskeletal remodeling, and nuclear positioning underscores the remarkable adaptability of DCs in traversing complex tissue landscapes.

This postulate was further reinforced by the work of Renkawitz et al. [80], who combined 3D‐collagen gels with microchannels to highlight that mDCs often navigate in complex geometries by selecting the path of least mechanical resistance. This selection depends on the spatial relationship between the nucleus and the microtubule‐organizing center (MTOC). When the nucleus leads, it acts as a mechanical probe to identify larger, more permissive pores. In contrast, when the MTOC leads, this probing mechanism is absent. Once the nucleus and MTOC pass through the largest pore, protrusions extending into smaller pores are retracted. Kopf et al. [81] showed that this retraction is MT‐dependent. MT plus ends are enriched in protrusions before MTOC passage but diminish in “loser” protrusions afterward, indicating MT destabilization precedes retraction. Photo‐induced MT depolymerization caused immediate protrusion collapse, confirming this causal link. Retraction was also associated with increased contractility, as nocodazole treatment elevated RhoA activity and myosin light chain phosphorylation. Inhibiting ROCK, a RhoA effector, partially restored directional persistence but not cell integrity. The RhoA GEF, GEF‐H1 (also known as Lfc), was identified as a key mediator linking MTs to actomyosin contraction, as previously described in other cell types [82]. GEF‐H1, normally sequestered by microtubules (MTs), is released upon MT depolymerization, thereby activating RhoA and promoting localized actomyosin contraction. Consistent with this mechanism, Duquesne et al. [83] recently demonstrated that kinesin‐1 coordinates cytoskeletal dynamics between the MT and actin networks in both iDCs and mDCs. Kinesin‐1 directly interacts with GEF‐H1, sequestering it and thereby preventing RhoA activation. In kinesin‐1–deficient DCs, this inhibitory interaction is lost, resulting in aberrant activation of GEF‐H1 and RhoA at the cell rear. Such mislocalized signaling enhances their migration speed but impairs nuclear deformation during passage through constricted environments, ultimately leading to inefficient migration in vivo.

Because both nucleus and MT organization and dynamics are key drivers of DC migration, Kroll et al. [84] studied the mechanism of how mDCs rapidly change their nucleus position, a process defined as nucleokinesis. Using microchannels, they found that nucleokinesis is more frequent when the nucleus starts in a wider pore but the cell chooses a narrower path. This process occurs in two phases: the nucleus first moves behind, then ahead of the MTOC. Surprisingly, MTs are not required for nucleokinesis, but Myosin II and actin are essential for maintaining the nucleus–MTOC axis. The nuclear–MTOC axis has also been dependent on external confinement: in dense environments, mDCs tend to relocate the nucleus behind the MTOC and organelles [53]. This is associated with a decrease of actin nucleation at the cell front, which relocates between the cell front and the nucleus, allowing cells to push against the surrounding environment and facilitate the nucleus's passage. This process was dependent on the cdc42 guanine exchange Dock8, and its downstream effector Wasp. The authors found a balance between the front actin pool and the central actin pool, that coordinates migration and path dilatation. Whether this coordination between front and perinuclear actin pools is similar to the one observed in iDCs under confinement and constriction [77] remains an open question. Interestingly, Weier et al. [85] reported that a fraction of mDCs arrest in G1 phase and accumulate multiple centrosomes. During migration, centrosomes cluster together, resulting in enhanced directional persistence toward a chemokine gradient, without altering migration speed, as compared to cells with a single centrosome.

Taken together, these studies collectively reveal that DC migration through confined environments is governed by a finely tuned interplay between nuclear mechanics and cytoskeletal dynamics. Of note, as DCs do not systematically choose the path of least mechanical resistance within tissues, it would be interesting to investigate how mechanical stimulation of DCs within narrow confining paths will affect their survival and immune function in vivo. This might be particularly relevant in DCs that infiltrate tumors, which often exhibit a very dense and stiff ECM.

### 3.3 Mechanosensing Is Not Restricted to Extracellular Constraints

While much work has been done to examine the impact of external physical constraints on DC properties, people have recently begun to examine the role of internal physical cues on phagocyte behavior and function. Ivanovska et al. [86] reported that small lipid droplets and artificial beads in macrophages cause nuclear indentation and even nuclear membrane rupture. As a professional phagocyte, iDCs ingest a variety of particles with varying physical properties (bacteria, parasites, apoptotic bodies). A recent preprint from Ruiz‐Fernandez et al. [87] shows that migrating mDCs reposition and deform intracellular parasitic cargo in a size‐dependent fashion using Myosin II, analogous to what occurs during nuclear deformation [77]. Interestingly, the repositioning of the parasitic vacuole behind the nucleus in a confined microchannel resulted in clear nuclear deformation, and increased vacuole size greatly decreased the DC migration speed, preventing successful cell passage through small pores. Other cargos have recently been shown to alter DC biology, specifically in the case of efferocytosis where ingested cell debris contributes to the cholesterol stores of DCs that are used to assemble cell‐surface lipid nanodomains that increase maturation marker expression and immune receptor signals [88]. Taken together, these works encourage further study investigating not only the impact of external constraints but also internalized cargos on the ability of DCs to exert their immune sentinel function.

## 4 Mechanosensing in Immature Dendritic Cells and Tissue Patrolling

A major function of iDCs is to patrol peripheral tissues by actively exploring their environment and allowing them to detect danger signals and invasive agents. This sensing process leads to DC maturation and initiates their migration toward draining lymph nodes. In this part, we will discuss what is known about how iDCs perceive surrounding mechanical cues, how their migration and antigen uptake capacity are powered by the dynamic remodeling of the actomyosin cytoskeleton, and how these processes—environmental sensing, locomotion and antigen uptake—are tightly coupled to optimize their immune surveillance function in time and space.

### 4.1 Membrane Protrusions and Adhesions in Mechanical Probing of Tissues by Immature DCs

iDCs can sense their environment through adhesions such as focal adhesion (Figure 3E) and podosomes (Figure 3F). Podosomes are dynamic, mechanosensitive actin‐rich structures formed of a 0.5–1 μm diameter core surrounded by an adhesive ring composed of integrins and adaptor proteins such as talin, vinculin, and integrin‐linked kinase (ILK) [89], which mediate strong and regulated interactions with the ECM. Actin polymerization and Myosin II contractility generate tension that stabilizes these structures by modulating mechanosensitive proteins, including integrins, vinculin, and paxillin. These macromolecular complexes contain proteins that unfold under mechanical strain, and thereby modulate many downstream signaling events, including a positive feedback loop that strengthens adhesions themselves [90]. Such adhesive structures display repeated protrusion and retraction cycles, at a single level but also collectively, and are critical not only for anchoring but also for mechanosensing and ECM remodeling. Van Den Dries. et al. [91] showed that DC podosomes are directly influenced by the spatial organization of the ECM. Using 2D‐ and 3D‐micropatterns, they found that their formation is not governed by substrate hydrophobicity properties; rather, cell adhesion itself is the sole prerequisite, and the available substrate area determines podosome density. On three‐dimensional (3D) micropatterned surfaces, DCs align podosomes along geometric edges, indicating a capacity for geometry sensing and adaptation to complex tissue topographies. Using high‐resolution electron and structured illumination microscopy, Gawden‐Bone et al. [92] revealed that podosomes in iDCs penetrate crosslinked gelatin gels, promoting localized degradation by the membrane‐bound metalloproteinase MMP‐14. Of note, matrix degradation and podosome protrusion are tightly coupled, enabling the uptake of matrix components. Several studies [93, 94, 95, 96] have unraveled the intricate architecture and dynamic regulation of podosomes, which relies on actin polymerization rather than Myosin II‐driven contractility [93, 96]. The model proposed is that growth of the podosome actin core generates tension, leading to recruitment of adaptor proteins such as Vinculin. While Myosin II is dispensable for this tension‐dependent recruitments, it might indirectly affect podosome length by controlling the disassembly of actin networks. Interestingly, substrate stiffness influences podosome dynamics [95] and their degradation capacity [94]. Stiffness has also been described to decrease the migration speed of immature DCs [97] on agarose gels through an unknown mechanism. Importantly, these studies were mainly performed on 2D surfaces rather than 3D ECM‐like substrate and it is unclear in which in vivo context they apply. Moreover, the role of the ECM composition—such as specific collagen subtypes, fibronectin, and hyaluronan—in modulating DC adhesion, podosome formation and antigen uptake remains largely unexplored.

Lamellipodia (Figure 3C) are protrusive structures enriched in branched actin that regulate cell‐matrix adhesion in fibroblasts [98]. Leithner et al. [99] have suggested, however, a putative role for lamellipodia in iDCs migrating in low‐adhesive conditions. *The authors observed that* inactivation of the WAVE complex in these cells leads to a striking cell shape change: iDCs lose their lamellipodia‐like ruffled and become elongated. This cell shape change results from decreased nucleation of branched actin by Arp2/3 in WAVE‐deficient DCs, whose actin network is dominated by parallel filopodia‐like actin filaments nucleated by Formins. As a result of these changes, WAVE‐deficient iDCs migrate faster and more directionally, and do not reorient toward chemotactic signals in collagen gels of complex geometries. Consistently, another study showed that Arp2/3 activated downstream of Cdc42 led to the formation of ruffles and macropinosomes (Figure 3C,D) at the front of iDCs [100], which can be used to uptake extracellular materials. Altogether, these results highlight that DC protrusions enable them probing both mechanical and chemical tissue properties and further impact on the migration and environment patrolling function.

Noticeably, macropinocytosis was further shown to confer DCs an unexpected property: the capacity to migrate under pressure. Indeed, unlike neutrophils that cannot follow paths exhibiting elevated hydraulic resistance [67], iDCs can do so. Using microfluidics devices, Moreau et al. [66] showed that this iDC property relies on the formation of macropinosomes that transport fluid from the cell front to the back. In other words, while fluid, which is incompressible, represents an obstacle for non‐macropinocytic cells, it does not for iDCs, as macropinocytosis makes them insensitive to hydraulic resistance. Accordingly, while neutrophils chose to follow paths of low hydraulic resistance, a process referred to as barotaxis, iDCs explore environmental paths independently of their hydraulic resistance. It was proposed that this behavior improves the capacity of iDCs to patrol complex tissue landscapes.

### 4.2 Calcium Channels and Migratory Space Exploration by immature DCs

Calcium influx is a central mediator that couples extracellular signals and mechanical stimuli to rapid cytoskeleton remodeling and actomyosin contraction. Although membrane mechanosensitive calcium channels such as Piezo have been implicated in DC maturation (as described later), their involvement in iDC migration remains largely uncharacterized. Nonetheless, several studies have highlighted important roles for intracellular calcium stores in the locomotion modes that iDCs use to explore their environment and achieve their immune sentinel function. Solanes et al. [101] showed that DCs exhibit intracellular calcium oscillations that are independent of extracellular calcium influx and associated with phases of fast and directional cell motility. These calcium spikes are regulated by IP_3_ receptors (IP_3_R), with the IP_3_R1 isoform playing a critical role. Mechanistically, IP_3_R1 activity supports the phosphorylation of Myosin II regulatory light chain (MLC), known to increase the activity of the motor protein at the back of iDCs. Accordingly, knocking down IP_3_R1 in these cells leads to reduced Myosin II activity and impaired directional migration, thereby limiting space exploration by iDCs. Interestingly, residual migration was observed in IP_3_R1,3‐silenced iDCs, suggesting the involvement of other calcium stores. Consistent with these results, it was highlighted that lysosomes locate at the back of migrating iDCs and release calcium through the channel TRPML1 (transient receptor potential cation channel, mucolipin subfamily, member 1) to locally enhance Myosin II activity and promote fast motility [72]. Interestingly, calcium release by TRPML1 also activates the lysosome‐associated transcription factor EB (TFEB), which is maintained in its inactive state at the surface of lysosomes thanks to phosphorylation by mTORC1. TRPML1 activation promotes TFEB translocation to the nucleus to maintain expression of genes involved in lysosome biogenesis including the *Trpml1* gene itself [102]. The TRPML1‐TFEB axis was further found to facilitate the migration of mDCs to lymph nodes in vivo. Interestingly, inhibition of macropinocytosis is sufficient to activate the TRPML1‐TFEB axis, suggesting that in iDCs, macropinosomes maintain mTORC1 activity by sensing and internalizing nutrients from the environment. This study showed an additional link between macropinocytosis and DC migration, which will further be developed below. It also revealed that lysosomes, which play a key function in antigen processing for MHC class II presentation, also can license mDCs for migration to lymph nodes, suggesting that these two processes might be coupled in time and space.

### 4.3 Optimization of Environment Sampling by Coupling Antigen Uptake to Migration in immature DCs

So far, we have explored how the role of actin protrusions and upstream regulators of actomyosin dynamics, such as calcium channels, enhances space exploration by iDCs. Here, we will describe how the regulation of the actomyosin cytoskeleton by CD74, also referred to as the MHC class II‐associated Invariant Chain (Ii), a molecule intimately linked to the antigen presentation function of DCs, also helps them optimize their migration trajectories and immune sentinel capacity. CD74 associates with MHC class II molecules during biosynthesis in the endoplasmic reticulum and guides them to endolysosomal compartments thanks to a di‐leucine motif localized in the CD74 cytosolic tail [103]. Once in endolysosomes, CD74 is degraded by cathepsins, and its last fragment CLIP is exchanged with peptides generated from extracellular antigens taken up by iDCs. Remarkably, it was found that CD74 also couples antigen uptake to DC migration by interacting with Myosin II [104]. This interaction diverts the motor protein from its migratory function localized at the cell rear to the cell front at the site of macropinosome formation, leading to migration slow‐down [105]. Contraction of this actomyosin network around macropinosomes promotes their transport toward the rear of the cell and delivery of antigens to endolysosomes. This process is followed by the disassembly of the network, Myosin II becoming again available for cell migration. Therefore, two actomyosin pools differently nucleated at the cell front or rear enable iDCs to couple antigen capture to cell migration [100]. Interestingly, it was shown using physical modeling that such an intermittent migration mode alternating fast and slow motility phases, might optimize the chances of iDCs to find rare antigens within the complex environment of tissues.

Altogether these studies highlight the many links existing between the capacity of iDCs to sample tissue antigens, (mechano)sense and move. Such links suggest the existence of adaptation mechanisms that might have emerged during evolution for better coordination of DC behavior with migration. In the next section, we will discuss the existence of analogous regulatory mechanisms in mature DCs, whose function is no longer to explore the environment but rather to efficiently migrate to lymph nodes to initiate adaptive immune responses.

## 5 The Interplay Between  Dendritic Cell Mechanosensing, Migration and Maturation

DC maturation and mechanosensing are deeply interconnected processes that continuously influence one another. DCs can be activated through a variety of environmental signals, which in turn shape their migratory behavior, adhesion dynamics, and overall sensitivity to mechanical cues. Conversely, the physical properties of the DC environment—such as dimensionality, stiffness, and confinement—can significantly impact DC maturation, altering their immune function and migratory strategies. In the following sections, we will first explore how distinct physical properties of the ECM impact DC maturation and migration. Then, we will examine how different signals leading to DC maturation trigger specific changes in their migration patterns.

### 5.1 Impact of Environmental Physical Cues on Dendritic Cell Immunogenic and Tolerogenic Maturation

The environmental physical properties that DCs experience during differentiation and maturation are highly diverse depending on the tissue where they reside and its inflammatory state [18]. It is essential to evaluate the contribution of these properties as they will ultimately influence the information mDCs will transfer to T cells during antigen presentation, and thus the type of adaptive immune response developed.

DCs have been reported that sensing the stiffness of their environment influences their sensitivity to other cues and affects later functions. It was shown that the expression of C‐lectin receptors such as CD206 and CD209 decreases in DCs plated on stiff substrates, suggesting that such a stiff environment might limit their ability to uptake microorganisms such as bacteria and yeast [106]. On the same line, Chakraborty et al. [107] have highlighted that bone marrow‐derived DCs (BMDCs) grown in a stiff environment that mimics fibro‐inflammatory diseases respond to Lipopolysaccharide (LPS) by enhancing inflammatory cytokine production, antigen‐presentation molecules expression, phagocytosis, and upregulation of glycolytic metabolism. In these cells, sensing of environmental stiffness is partly mediated by the Piezo1 stretch‐activated calcium channel, which activates the transcription factor TAZ. Consistently, treatment of DCs with a Piezo1 agonist can increase the production of inflammatory mediators on soft substrates only in the presence of TAZ [107]. In addition, LPS‐treated DCs knockout for Piezo1 upregulate the production of immunosuppressive cytokines such as TGFβ on stiff substrates, whereas the expression of inflammatory cytokines such as IFNγ is decreased [108]. Interestingly, at steady state in aged mice, Piezo1‐deficient DCs show fewer IFNγ‐expressing Th1 cells but more Foxp3^+^ Tregs, indicating a skewing of immune responses toward tolerance. Similar results were observed in cancer models in mice, with decreased tumor recognition by the immune system and favored tumor growth [108].

Sensing of tissue porosity has also been uncovered as central in controlling DC maturation. DCs migrating in peripheral tissues and to lymph nodes undergo pronounced shape changes due to physical constraints present within the microenvironment of tissues [77, 109]. These large cell deformations are sufficient to trigger iDCs maturation and their migration to lymph nodes, which involves the activation of the lipid metabolism enzyme cPLA_2_ (cytosolic phospholipase A_2_) [78]. Cell confinement or hyper‐osmotic shock has been shown to activate this enzyme by increasing nuclear envelope tension, which triggers cPLA_2_ insertion into this membrane and the production of arachidonic acid (AA) [110, 111, 112]. This lipid mediator can be transformed into eicosanoids by different enzymes. Prostaglandins and thromboxane are generated from AA by cyclo‐oxygenases, and leukotrienes are produced by 5 lipo‐oxygenases [113]. iDC confinement [114] at a precise height (3 μm), which allows nuclear membrane tensioning without rupture, triggers the appearance of a perinuclear network of branched actin nucleated by Arp2/3 and subsequent cPLA_2_ activation [78]. This has two effects: (1) it increases within seconds the migration speed of DCs by increasing cortical actomyosin contractility, and (2) it leads later on to the production of PGE_2_ that activates the IKKβ‐NFκB axis and reprograms DC transcription into mature CCR7^+^ DCs. In vivo this is sufficient to license DCs for migration toward lymph nodes in the absence of inflammation, as evidenced by decreased numbers of DCs in skin‐draining lymph nodes from conditional cPLA_2_ knockout mice at steady state. Interestingly, this phenotype mainly concerns cDC2s, which had indeed been shown to migrate more than DC1s from the skin to the draining lymph node under homeostatic conditions. Of note, although these mechanically mature DCs express CCR7, they display a drastically distinct transcriptional program as compared to LPS‐mature DCs, with low expression of genes associated with DC maturation such as type I Interferon Stimulated Genes and elevated expression of anti‐inflammatory cytokines such as IL‐10. This is consistent with mechanical DC maturation producing tolerogenic DCs that maintain tolerance in homeostasis. Accordingly, only mechanically mature—but not LPS‐mature DCs—required cPLA_2_ to reprogram their transcription. Whether these two DC maturation pathways are or are not exclusive remains an open question. This study highlights the capacity of DCs to use their actin cytoskeleton and nucleus to sense the geometry of the environment. It further stresses the importance of tissue physical properties in tuning the ability of these cells to balance tolerance and immunity.

### 5.2 Impact of Dendritic Cell Maturation on Their Migration Mode and Environment Mechanosensing

As DCs become mature, they reduce their macropinocytic activity and increase the expression of surface molecules and chemokine receptors that support their migration to lymph nodes. A key component of this process is the upregulation of the chemokine receptor CCR7, which enables DCs to detect the chemokine CCL21 produced by lymphatic vessels [115]. This interaction guides DCs toward draining lymph nodes, where they present antigens to T cells and eventually initiate immune responses. In addition to these molecular changes, DC maturation also decreases their adhesive properties through cytoskeleton reorganization. West et al. [116] and van Helden et al. [117] described how DC maturation turns on distinct but converging pathways that trigger the disappearance of their adhesion structures. Toll‐like receptor (TLR) 4 signaling induces podosome disassembly through metalloprotease ADAM17 [116]; unlike prostaglandin E2 (PGE2), which induces podosome dissolution through EP2 and EP4 receptors, leading to activation of the RhoA–ROCK–Myosin II axis. PGE2 stimulation also suppresses Rac1 and Cdc42 activity, further decreasing cell adhesion and reinforcing actomyosin contractility [117]. Of note, these studies were performed in vitro on 2D substrates, which do not reflect the complexity of the ECM in vivo. Anyhow, they strongly suggest that the transition from iDCs to mDCs might increase cell motility at least in part by decreasing cell adhesion and exertion of traction forces on the environment.

Consistent with these findings, Vargas et al. [100] reported that the actin cytoskeleton is deeply reorganized upon TLR4 activation of DCs by LPS, independently of CCR7 expression. LPS‐DCs reduce CDC42‐dependent actin nucleation of branched‐actin networks by Arp2/3 at the cell front and increase RhoA‐1 (Formin)‐dependent actin bundles at the cell rear. These results were confirmed in vivo, with a decreased migration of mDia1^−/−^ DCs to the lymph node. This mechanism contributes to decreased macropinocytosis but also enhances the migratory capacity of mDCs. Decreased macropinocytosis in mature DCs makes them sensitive to hydraulic resistance [66] as suggesting that they could use this environmental cue to optimize their path through lymphatic vessels [118] and efficiently reach lymph nodes to initiate adaptive immune responses.

Noticeably, several studies have revealed that mDCs exhibit increased cellular stiffness and decreased membrane fluidity, which may support their transit through tissue barriers. Bufi et al. [12] demonstrated that human myeloid antigen‐presenting cells, including DCs, exhibit distinct viscoelastic properties that are modulated by inflammatory signals. Using single‐cell rheometry, they found that mDCs become stiffer than their immature counterparts, a change driven by increased actin polymerization and myosin II activity. DC stiffening is independent of classical maturation markers and depends on the maturation signal used (IFNy, LPS, TNFα+PGE2). Blumenthal et al. [119] further showed that DC maturation leads to a 2–3‐fold increase in cortical stiffness, primarily through Wasp and Arp2/3‐dependent increase of actin polymerization. Increased DC stiffness enhances their capacity to prime naïve CD4+ T cells by lowering the threshold of antigenic stimulation required for activation. Thus, DC stiffness acts as a biophysical costimulatory signal, complementing molecular cues at the immunological synapse. Leblanc‐Hotte et al. [120] introduced interferometric deformability cytometry to characterize intracellular mechanical properties of bone marrow‐derived DCs. Their findings confirmed that maturation induces DC stiffening but reduces nuclear mobility under fluidic stress. Collectively, these studies show that DC maturation is not solely a biochemical but also a mechanical process.

## 6 Conclusions and Perspectives

**FIGURE 4:**
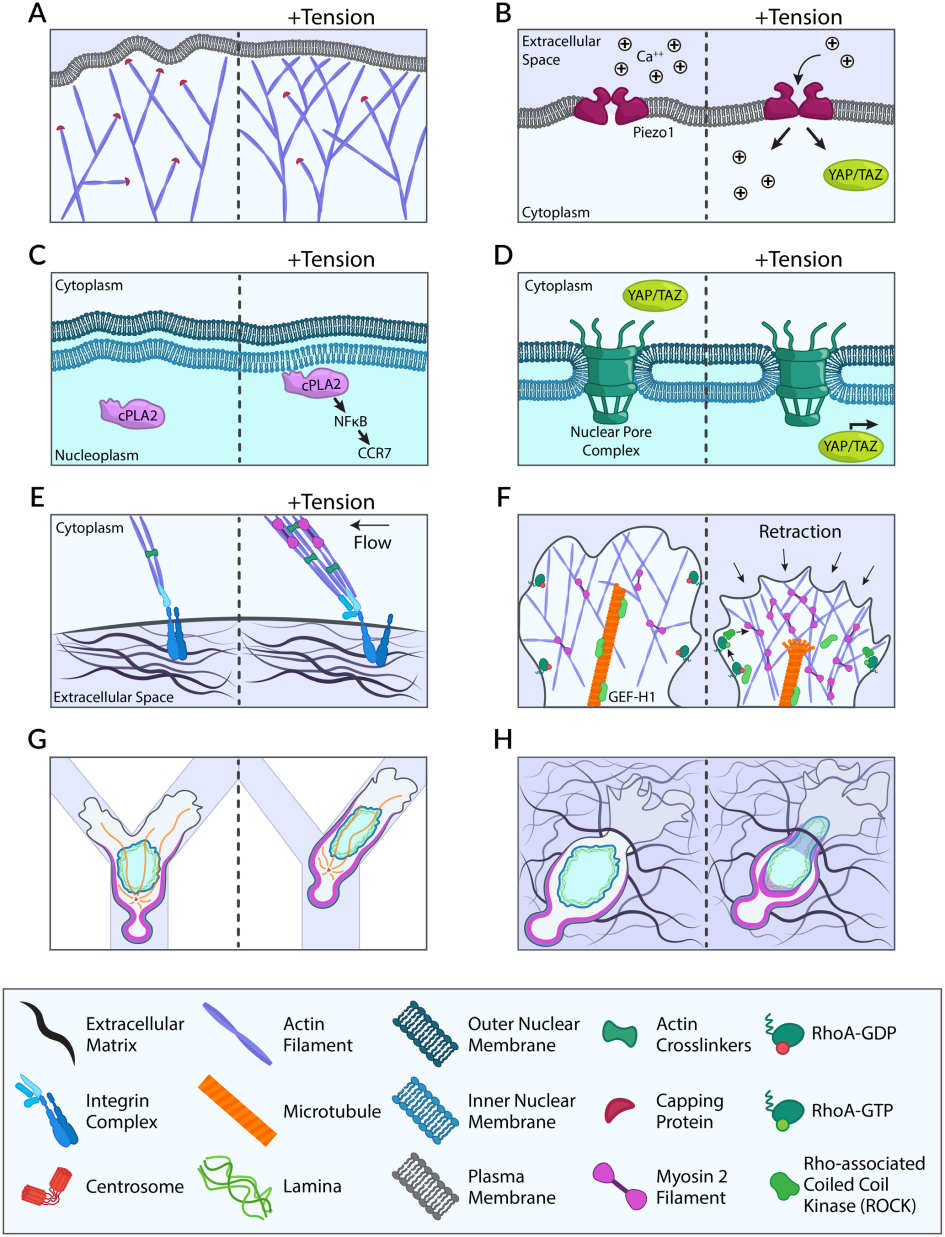
Mechanosensing mechanisms in dendritic cells. (A) Low plasma membrane tension promotes cortical branched actin assembly, stabilizing protrusions; increased membrane tension subsequently suppresses actin polymerization. (B) Elevated membrane tension activates mechanosensitive calcium channels (Piezo1), leading to calcium influx and downstream signaling pathways such as YAP/TAZ. (C) Under confinement, Arp2/3 mediated nuclear envelope tension activates cPLA2, driving eicosanoid production and CCR7 upregulation through the PGE2‐NFκB pathway. (D) NE tension also stretches nuclear pore complexes, enabling YAP/TAZ nuclear translocation and transcriptional regulation. (E) The cytoskeleton interacts with the extracellular matrix through integrin‐mediated adhesions; actomyosin contraction generates traction forces that promote locomotion. (F) Local microtubule depolymerization releases GEF‐H1, which activates RhoA via phosphorylation, enhancing actomyosin contractility through ROCK activity and driving plasma membrane retraction. (G) Nuclear deformability and the nucleus–MTOC axis sense spatial constraints, directing dendritic cell migration along paths of least resistance. (H) Actin polymerization in front of and around the nucleus facilitates nuclear squeezing and ECM deformation, enabling migration through confined spaces.

It is now widely recognized that the cytoskeleton not only dictates cell morphology and shape but also acts as a critical sensor of the diverse mechanical cues encountered within tissues. These cues vary greatly in rigidity, three‐dimensional organization, topography, adhesion, and hydraulic resistance, reflecting the heterogeneity of ECM composition and organization across tissues. In DCs, early work from the Mellman lab revealed mechanosensitivity shortly after their discovery [121, 122], yet the underlying mechanisms have only recently come to light thanks to recent developments in the field of mechanobiology. As summarized in Figure 4, we here provide multiple pieces of evidence showing that DC mechanosensing involves both actin and microtubule cytoskeletons, as well as the nucleus, that is, the stiffest organelle in the cell. Beyond guiding DC migration through complex environments and toward lymph nodes, mechanosensing profoundly influences DC immunogenicity versus tolerogenicity [78]. These findings underscore that the physical properties of tissue microenvironments might shape DC capacity to mount immune responses and limit chronic inflammation.

This opens exciting perspectives for exploring DC mechanosensing in pathological contexts such as fibrosis and cancer, where ECM is extensively remodeled and tissue physical properties are altered. Interestingly, recent work highlights that in late‐stage tumors, DC migration to lymph nodes is impaired because of decreased actomyosin contractility [123]. Whether such downregulation of DC migration by the tumor microenvironment involves mechanosensing is an interesting hypothesis to be explored. Similarly, it was recently shown that plasmacytoid DCs respond to skin stiffness by decreasing interferon production through activation of the transcription factor NRF2 [124], linking mechanosensing to metabolic adaptation. Whether such mechanisms extend to conventional DCs remains unclear. Future research should aim to dissect how distinct DC subsets integrate mechanical and biochemical signals to fine‐tune immune responses and how such integration shapes their ability to recognize, transport, and present antigens to lymphocytes. Understanding these processes will not only illuminate fundamental aspects of DC biology but also inform therapeutic strategies targeting immune dysfunction in diseases including fibrosis and cancer.

## Funding

This work was supported by Agence Nationale de la Recherche, InfEx (ANR‐20‐ce15‐0023) to HDM, European Research Council, SHAPINCELLFATE to AMLD and MP, and Labex DCBIOL (ANR‐10‐IDEX‐0001‐02) to PSL. This work has received support under the program France 2030 launched by the French Government.

## Conflicts of Interest

The authors declare no conflicts of interest.

## Data Availability

No new data were generated or analyzed in support of this review.
